# The ischaemic and scar burden measured by cardiac magnetic resonance imaging in patients with ischaemic coronary heart disease from the CE-MARC study

**DOI:** 10.1186/1532-429X-15-S1-O105

**Published:** 2013-01-30

**Authors:** Sven Plein, Bernhard A Herzog, Neil Maredia, Ananth Kidambi, Manish Motwani, Akhlaque Uddin, David P Ripley, Catherine J Dickinson, Julia Brown, Jane Nixon, Colin Everett, John P Greenwood

**Affiliations:** 1 Multidisciplinary Cardiovascular Research Centre & Leeds Institute of Genetics, Health and Therapeutics, University of Leeds, Leeds, UK; 2 Leeds Teaching Hospitals NHS Trust, Leeds, UK; 3 University of Leeds, Leeds, UK

## Background

The prognostic importance of the ischaemic and scar burden, and their impact on coronary heart disease (CHD) patient management is well established from single photon emission computed tomography (SPECT) studies. Recently, cardiac magnetic resonance (CMR) has been shown to have superior sensitivity for the detection of CHD compared with SPECT [1]. However, the ischaemic and the scar burden measured by CMR and SPECT have not been compared.

## Methods

From the prospective CE-MARC study, all patients who had significant coronary artery stenosis (≥70% of a first order coronary artery or ≥50% of the left main artery) on quantitative invasive coronary angiography and ischaemia on both CMR and SPECT were selected. The summed stress score (SSS), the summed rest score (SRS) as well as the summed difference score (SDS) were assessed based on a 5-point scoring scale (0=normal; 4=severe) for perfusion defects and/or late gadolinium enhancement (LGE) using a 16-segment model; comparisons were made between the two modalities. Bland-Altman limits of agreement (BA) were calculated.

## Results

**Figure 1:**
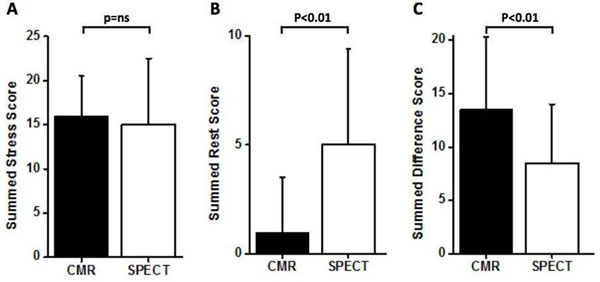


One-hundred-and six of the 752 CE-MARC patients fulfilled the inclusion criteria for this analysis. The median SSS was similar between CMR and SPECT (median ± interquartile range: 16±9 vs. 15±15, p=ns; Fig. 1A). The median SRS was significantly lower (1.6±3.9 vs. 12.2±10.7, p<0.01; Fig. 1B) and the median SDS significantly greater by CMR than by SPECT (13.5±6.8% vs. 8.5±5.5%, p<0.01; Fig. 1C). Overall there was moderate correlation and agreement (SSS: r=0.36, BA= -22.0 to 21.7; SRS: r=0.42, BA= -7.9 to 15.1; SDS: r=0.30, BA= -21.1 to 15.4).

## Conclusions

CMR is an alternative to SPECT in identifying the presence of CHD. This subanalysis of CE-MARC shows that for the assessment of overall disease burden, the two tests are comparable. However, there is a discrepancy in the detection of ischaemia versus scar between the two methods and CMR measures significantly less scar but significantly more ischaemia than SPECT. Potential reasons for this discrepancy include the differences in the methodology for scar imaging (LGE vs. matched defect) and the difference in cardiac coverage for perfusion assessment. Further studies will have to show the impact of these findings on patient outcome.

## Funding

The CE-MARC study was funded by a British Heart Foundation Programme Grant (RG/05/004). S.P is funded by British Heart Foundation fellowship (FS/10/62/28409).

## References

[B1] Wagner GA J Cardiovasc Magn Resn 2009

